# Anti-programmed Cell Death Protein-1 Therapy in Intrahepatic Cholangiocarcinoma Induced Type 1 Diabetes: A Case Report and Literature Review

**DOI:** 10.3389/fpubh.2022.917679

**Published:** 2022-06-16

**Authors:** Zhi-Kai Zheng, Jiong-Liang Wang, Wen-Xuan Li, Tian-Qing Wu, Min-Shan Chen, Zhong-Guo Zhou

**Affiliations:** ^1^Department of Liver Surgery, Sun Yat-sen University Cancer Center, Guangzhou, China; ^2^Collaborative Innovation Center for Cancer Medicine, State Key Laboratory of Oncology in South China, Guangzhou, China

**Keywords:** immune checkpoint inhibitors, anti-programmed cell death protein-1, immune-related adverse events, type 1 diabetes, intrahepatic cholangiocarcinoma

## Abstract

Immune checkpoint inhibitors, widely used in the treatment of malignancies, can improve the prognosis of patients, while it also can induce various immune-related adverse events, and type 1 diabetes induced by anti-programmed cell death protein-1 is a rare but severe complication. Here we reported a case of type 1 diabetes induced by anti-PD-1 which was to treat intrahepatic cholangiocarcinoma. The case was a 61-year-old female who developed diabetes and ketoacidosis symptoms at the 16th week after anti-PD-1 therapy. Her blood glucose was 30.32 mmol/L, HBA1c was 8.10%, and C-peptide was <0.10 ng/ml. The patient was diagnosed as fulminant type 1 diabetes mellitus complicated with ketoacidosis induced by anti-PD-1, and was treated with massive fluid rehydration, intravenous infusion of insulin and correction of acid-base electrolyte disorder. Hepatectomy was performed after stabilization, and the patient was treated with long-term insulin. Through the case report and literature review, this study aims to improve oncologists' understanding of anti-PD-1 induced type 1 diabetes, so as to make early diagnosis and treatment of the complications and ensure medical safety.

## Introduction

Immune checkpoint inhibitors (ICIs), which have been widely applied to patients with advanced malignancies, can improve the prognosis of patients (1–3). It can enhance the anti-tumor immune response by blocking immune checkpoints (ICs) (4). As the representative of ICIs, anti-programmed cell death protein 1 (anti-PD-1) has been studied in animals and in several clinical trials. It was approved by the Food and Drug Administration (FDA) for the treatment of types of cancers, such as squamous cell carcinoma of head and neck, non-small cell lung cancer, renal carcinoma, urothelial carcinoma, melanoma, Hodgkin's lymphoma, liver cancer, colorectal cancer and so on (5, 6). In addition, immunotherapy can also be used for other advance solid tumors with limited therapies, such as intrahepatic cholangiocarcinoma, and it has achieved great success (7, 8).

However, ICIs can induce various immune related adverse events (irAEs) including pneumonitis, hepatitis, colitis, dermatitis and endocrinopathies (9, 10). Endocrinopathies are common complications, including diabetes mellitus, hypophysitis, thyroid dysfunction, primary adrenal insufficiency (11). Although the incidence of immune checkpoint inhibitor-induced diabetes is low, it may lead to life-threatening diabetic ketoacidosis. So it's necessary for us to understand the clinical manifestation, examination and therapies of the immune checkpoint inhibitor-induced diabetes. In this report, we present a patient with intrahepatic cholangiocarcinoma who had anti-PD-1-induced type 1 diabetes, in order to improve oncologists' understanding of type 1 diabetes induced by anti-PD-1.

## Case Presentation

The patient, a 61-year-old female with no diabetes mellitus history and family history of diabetes, whose HBsAg, HBeAb, and HBcAb were all positive while the anti-HCV was negative, was diagnosis as intrahepatic cholangiocarcinoma by pathology in March 25th, 2021. However, the hepatic mass was unresectable. Hepatic arterial infusion chemotherapy (FOLFOX protocol: Oxaliplatin 130 mg/m^2^, Calcium levofolinate 200 mg/m^2^, 5-fluorouracil 400 mg/m^2^) combined with anti-PD-1 immunotherapy (Tislelizumab 200 mg) was performed from March 25, 2021 to June 17, 2021 for 3 cycles that every 3 weeks was a cycle. No abnormal blood glucose and HBA1c were detected during medication. After that the Magnetic resonance imagination and CT examination on July 8, 2021 showed that the hepatic mass was smaller than before, which meant that the patient was admitted to the hospital for liver cancer resection. However, on the second day after admission, the patient felt thirsty, nauseous and somnolent. The laboratory test (Table 1) was as following: glucose: 30.32 mmol/L, HBA1c: 7.6%, pH: 7.18, PaCO_2_: 26.6 mmHg, HCO_3_-: 9.7 mmol/L, lactic acid: 2.9 mmol/L, urinary glucose: 4+, urinary ketone: 4+, GAD: -, ICA: -, IAA: -, IA-2A: -. Considering her clinical manifestation and examination, type 1 diabetes accompanying with ketoacidosis induced by anti-PD-1 was diagnosed. Intravenous massive fluid infusion, infusion of insulin and correction of acid-base electrolyte disorder were given. After that, the diabetes related laboratory test (Table 2) was reexamined and as following: Blood glucose: 20.14 mmol/L, HBA1c: 7.60%, insulin (0 h): 9.03 mU/L, (1 h) 55.34 mU/L, (2 h) 37.52 mU/L, C-peptide (0 h): 0.09 ng/ mL, (1 h): 0.08 ng/ mL, (2 h): 0.08 ng/ml. After consultation with the endocrinology department, the insulin dose was adjusted for several times. Finally, insulin aspartate (4iu in the morning, 6iu at noon, 8iu in the evening I.H. TID) and insulin glargine (10iu at 10 PM I.H. QN) were administered, and the control of blood glucose was stable. After 2 weeks, the patient was admitted to the hospital again for intrahepatic cholangiocarcinoma resection, and the operation was successful. One week after surgery, the patient was discharged safely and insulin was continued to treat diabetes. One month after the operation, the patient returned to the hospital for further consultation. There was no abnormality in liver tumor-associated antigen and blood glucose and no tumor recurrence showed by magnetic resonance imagination. However, the patient was dependent on insulin therapy.

**Table 1:** The laboratory test.

**Laboratory test**	**Value**	**Reference**
**Complete blood count**		
White blood cell (×109/L)	11.37	3.5–9.5
Red blood cell (×1,012/L)	4.11	3.8–5.1
Hemoglobin (g/L)	131	115–150
Platelet (×109/L)	137	100–300
Neutrophil (%)	86.4	40–75
Lymphocyte (%)	6.7	20-50
**Blood biochemistry**		
AST (U/L)	18.8	13–35
ALT (U/L)	15.7	7–40
Creatinine (umol/L)	72	41–81
Serum urea (mmol/L)	8.5	3.1–8.8
Blood glucose (mmol/L)	30.32	3.9–6.1
HBA1c (%)	7.6	3.6–6.0
Free triiodothyronine (pmol/L)	2.81	2.80–7.10
Free tetraiodothyronine (pmol/L)	15.70	12.00–22.00
Thyroid stimulating hormone (uIU/ml)	0.80	0.27–4.20
Anti-thyroid peroxidase (U/ml)	17.3	0–35.00
Thyroglobulin (ng/mL)	10.6	3.50–77.00
Human growth hormone (ng/mL)	2.72	0.126–9.88
Follicle-stimulating hormone (mIU/mL)	121.3	Menopause 25.8–134.8
Luteinizing hormone (mIU/mL)	41.28	Menopause 7.7–58.5
Prolactin (uIU/mL)	434.60	72–511
**Blood electrolytes**		
Na+ (mmol/L)	127	137–147
K+ (mmol/L)	4.68	3.5–5.3
Cl- (mmol/L)	95.1	99–110
Ca2+ (mmol/L)	2.88	2.11–2.52
**Arterial blood gas**		
pH	7.18	7.35–7.45
PaO_2_ (mmHg)	98.2	80–108
PaCO_2_ (mmHg)	26.6	35–45
Standard bicarbonate (mmol/L)	11.7	23.3–24.8
Actual bicarbonate (mmol/L)	9.7	22–27
Base excess (mmol/L)	−17	−3–3
Anionic gap (mmol/L)	26.8	12–20
Lactic acid (mmol/L)	2.9	0.7–2.5
**Urina**
Urinary occult blood test	-	-
Urinary glucose	4+	-
Urinary ketone	4+	-
**Diabetes related autoantibodies**		
Glutamic acid decarboxylase antibody	-	-
Islet-cell antibody	-	-
Insulin antibody	-	-
Anti-insulinoma antigen-2 antibody	-	-

**Table 2:** The reexamined laboratory test.

**Diabetes related laboratory test**		**Value**
Blood glucose (mmol/L)		20.14
HBA1c (%)		7.6
insulin (mU/L)	0 h	9.03
	1 h	55.34
	2 h	37.52
C-peptide (ng/ mL)	0 h	0.09
	1 h	0.08
	2 h	0.08

## Discussion

### The Incidence of Anti-PD-1 Diabetes

Anti-PD-1 induced diabetes is a relatively rare complication, whose incidence ranges from 0.2 to 2% (1, 2, 12–15). The meta-analysis by Akturk et al. (16) and the study by Youssef et al. (15) both indicated a current incidence of 0.9%. In addition, different kinds of anti-PD-1 lead to different rates of diabetes. Several studies showed that the incidence of diabetes induced by Nivolumab is 0.9%, higher than that of Pebolizumab (0.2%) (9, 17, 18). Due to the loss of blood glucose monitoring and the inobvious early symptoms of the disease, the reported incidence is lower than the actual. And as the anti-PD-1 is initiated at an earlier disease stage and to a greater extent, the incidence of immunotherapy-related diabetes will increase gradually (1, 4).

### The Pathogenesis of Anti-PD-1 Diabetes

PD-1 receptor is a type 1 transmembrane glycoprotein with a molecular weight of 50–55 kDa, belonging to the immunoglobulin superfamily, whose ligands are PD-L1 and PD-L2 (19, 20). The mechanism of anti-PD-1 leading to diabetes is still unclear, and it may be associated with activated T cells. PD-1 exists on the surface of T cells, and it can suppress the activation and proliferation of T cells through inhibiting the PI3K/Akt pathway to block the uptake of glucose and gluconeogenesis by binding with ligands on the surface of immune cells, islet cells and other cells, which results in immune tolerance (11, 18–23). By blocking the PD-1/PD-L1 and PD-L2 pathways, anti-PD-1 blocks the inhibitory signal of T cells, leading to the proliferation and activation of islet cell-specific T cells, which leads to the destruction of islet cells and the occurrence of diabetes (21, 24). This has been demonstrated in several mouse models (2, 15, 20, 21, 25). The mechanism of anti-PD-1 diabetes still needs further study.

### The Clinical Manifestations of Anti-PD-1 Diabetes

Similar to traditional type 1 diabetes, diabetes induced by anti-PD-1 may present symptoms and signs of hyperglycemia, such as polyuria, polydipsia, polyphagia (20). Some patients may be accompanied with diabetic ketoacidosis (DKA), which presents as nausea, vomiting, abdominal pain, dyspnea, somnolence and even coma (3). The incidence of DKA is very high, ranging from 62.1 to 81% (11, 13, 16, 21, 23). DKA is always the reason for diagnosis, which greatly increases the risk of death, which is also the reason why physicians need to be vigilant against diabetes induced by anti-PD-1 (11). In addition, patients may have no symptoms and be diagnosed with hyperglycemia only by occasional blood glucose monitoring. In this case, the patient's symptoms were obvious and were successfully controlled after active treatment, which indicates the importance of early identification and therapy of diabetes.

### The Laboratory and Imaging Examination of Anti-PD-1 Diabetes

In order to timely identify anti-PD-1 diabetes and prevent the occurrence of ketoacidosis, it is necessary to find some relative biomarkers or indicators.

#### Blood Glucose

The blood glucose levels of patients with anti-PD-1 diabetes were elevated, which were 33.4 ± 11.5 (13.7–67.3) mmol/L [602 ± 207 (246–1,211) mg/dl] (16). The time to onset varied from 1 to 52 weeks (10, 11, 13, 14, 16, 18, 26). In this case, the patient's blood glucose level was very high at onset (30.32 mmol/L), and the onset time was 16 weeks after treatment with anti-PD-1, which was consistent with literature reports. Due to the uncertainty of blood glucose level, onset time and high incidence of DKA, blood glucose monitoring must be part of routine monitoring before and during anti-PD-1 treatment (4, 20). As for the frequency of monitoring, Akturk et al. (16) pointed out that 71% of patients developed diabetes within 3 months after anti-PD-1 treatment, so the frequency of blood glucose detection should be appropriately increased at the beginning of treatment and gradually decreased after 3–4 months (18).

#### Hemoglobin A1c

Hemoglobin A1c (HbA1c), a measure of average blood glucose over the preceding 3 months, is a common indicator for diabetes monitoring. Different from traditional diabetes, HbA1c levels in patients with anti-PD-1 diabetes were mostly close to normal or slightly elevated, ranging from 5.8 to 11.4% (1, 4, 5, 18, 21, 22, 27). In this case, the patient's HbA1c was 7.6%. Since the acute onset of diabetes induced by anti-PD-1, the early HbA1c level is mostly normal, which might not be a good screening indicator (11). Nevertheless, continuous monitoring of HbA1c is significant for patients to minimize the risk of diabetes development and adjust the treatment regimen (15, 22). Routine monitoring of HbA1c after anti-PD-1 treatment is still of importance.

#### C-Peptide

C-peptide, which is secreted by islet β cells, is an indicator of islet β cell function and is not influenced by exogenous insulin. In patients with anti-PD-1 diabetes, C-peptide levels might be low or even absent, which suggests the rapid destruction of β cells (1, 3, 4, 14, 21, 22). A retrospective study in 2021 indicated that the patients' C-peptide level were 0.1 ng/mL (0.012–3.16 ng/mL) (18). In this case, multiple measurements of C-peptide were all <0.09 ng/ml. According to the definition of fulminant type 1 diabetes in Japan, C-peptide is one of the important indicators (21). The diagnostic criteria (28) are as follows: hyperglycemia develops into ketosis or ketoacidosis within 1 week; Blood glucose ≥16.0 mmol/L and HbA1c <8.5% at first diagnosis; Urinary C-peptide <10 μg/d or fasting serum C-peptide <0.3 ng/mL and postprandial C-peptide <0.5 ng/mL. As an important diagnostic indicator of fulminant diabetes, C-peptide should be closely monitored, especially for patients with rapidly elevated blood glucose (22).

#### Diabetes-Related Autoantibodies

For patients with autoimmune diabetes, diabetes-related autoantibodies, including glutamic acid decarboxylase antibody (GADA), islet-cell antibody (ICA), insulin antibody (IAA), anti-insulinoma antigen-2 antibody (IA-2A), and zinc transporter 8 autoantibody (ZnT8), are usually tested (22). Among patients with anti-PD-1 diabetes, 50–56% were detected at least one diabetes-related autoantibody, and the most prevalent were GADA (1, 4, 5, 9, 11, 16). Chang et al. (20) pointed out that autoantibody detection may not be useful as a biomarker to predict the occurrence of anti-PD-1 related diabetes. Patients could have autoantibody before or after anti-PD-1 treatment, or turn from positive to negative after treatment. However, Gauci et al. (21) found that autoantibodies were associated with the length of time interval between the onset of autoimmune diabetes. GADA-positive patients had a shorter median interval between onsets than negative ones (1, 4, 5, 9, 16, 26, 29). Akturk et al. (16) also pointed out that autoantibody positive patients were more likely to develop DKA (86 vs. 60%; *P* = 0.02). Therefore, diabetes-related autoantibodies are useful biomarkers and should be detected when necessary.

#### Pancreatic Exocrine Enzyme and Pancreatic Imaging Examination

20–52% of patients with anti-PD-1 diabetes had elevated lipase or amylase, suggesting inflammation of the pancreas, which may be related to destruction of islet β cells (13, 30). In addition, some scholars observed pancreatic enlargement before the onset of diabetes and progressive pancreatic atrophy afterwards, which may also be used as an indicator for early diagnosis or monitoring (14, 27). However, some scholars reported normal or no significant changes in pancreatic imaging (20). Further studies are needed to understand the relationship among pancreatic exocrine enzyme, imaging and anti-PD-1 diabetes.

#### Diabetes Related Gene Test

The development of anti-PD-1 diabetes is also related to the susceptibility of genes. Among the patients whose HLA genotypes were tested, 61–76% had genotypes related to ICIs-induced diabetes, much higher than the normal population, and *HLA-DR4* was the dominant one (6, 13, 15). *HLA-DR4* can be used as a risk predictor of type 1 diabetes induced by anti-PD-1 (2). As HLA genotypes are expensive and time-consuming, their potential as predictors remains to be further studied (15, 18). The author believes that high-risk HLA testing should be examined on patients to be treated with anti-PD-1 to evaluate the risk of diabetes if permission.

### The Diagnosis of Anti-PD-1 Diabetes

In this case, the patient had no previous history of diabetes, and the level of blood glucose was normal before treatment. After three cycles of anti-PD-1 therapy, the blood glucose was 30.32 mmol/L, HbA1c was 7.6%, and fasting and postprandial C-peptide were all <0.1 ng/ mL, suggesting rapid destruction of pancreatic β cells. In addition, the patient developed symptoms of diabetic ketoacidosis rapidly after hyperglycemia, so the diagnosis was considered as fulminant type 1 diabetes induced by anti-PD-1 therapy. The patient was also treated with hepatic arterial infusion chemotherapy. According to relevant literature reports, oxaliplatin and 5-fluorouracil may also induce diabetes (23, 31–33). However, oxaliplatin and 5-fluorouracil damage islet β cells through damaging the pancreas or causing pancreatic inflammation, which would influent insulin's synthesis and secretion, finally resulting in abnormal blood glucose regulation (31–35). In this patient, we adopt hepatic artery infusion chemotherapy (FOLFOX protocol: Oxaliplatin 130 mg/m^2^, Calcium 48 levofolinate 200 mg/m^2^, 5-fluorouracil 400 mg/m^2^) which lasted 48 h so that oxaliplatin and 5-fluorouracil could not reach the pancreas or the dose was very small which may not lead to diabetes. At the same time, the chemotherapy-induced diabetes reported by the relevant literature was all caused by systemic chemotherapy, and there were no reports on hepatic artery infusion chemotherapy. Therefore, the possibility of oxaliplatin and 5-fluorouracil inducing diabetes is basically ruled out.

### The Therapy and Prognosis of Anti-PD-1 Diabetes

For the treatment of diabetes induced by anti-PD-1, 71 patients in 56 literatures reviewed by Akturk et al. (16) all required continuous insulin treatment afterwards. Among the 66 patients reported by Chang et al. (20), only one patient stopped insulin treatment 54 days after the onset of diabetes. Autoimmune diabetes caused by anti-PD-1 is irreversible and is associated with massive destruction of islet β cells (3, 18). These patients require long-term insulin to control blood glucose, and continuous insulin treatment is the standard treatment (18, 22, 23). This case required long-term insulin therapy, too.

As an autoimmune disease, whether to use glucocorticoids is controversial. Although some clinicians attempted to use glucocorticoids to salvage islet function, unlike other autoimmune diseases, glucocorticoids therapy had no effect, which may be related to the complete destruction of β cells (14, 36). Chang et al. (20) reviewed the cases of systemic hormone therapy and noted that none of the patients were able to reverse diabetes. Moreover, glucocorticoid treatment may lead to deterioration of blood glucose and insulin resistance in some patients (1, 10, 22). Therefore, hormone therapy is not recommended for the patient and was not used in this case.

Whether to continue anti-PD-1 treatment and when to continue treatment also depends on the severity of the patient's diabetes. According to the criteria of tumor treatment-related adverse events, anti-PD-1-induced diabetes can be divided into five grades according to severity: Grade 1, asymptomatic or mild symptoms and fasting blood glucose >7 mmol/L (126 mg/dL); Grade 2, moderate symptoms and fasting blood glucose >8.9 mmol/L (160 mg/dl); Grade 3, severe but not life-threatening symptoms, requiring hospitalization; Grade 4, life-threatening symptoms requiring emergency intervention; Grade 5, death (11). For grade 1 patients, anti-PD-1 therapy can be continued; Patients with grade 2–3 need to stop anti-PD-1 and receive insulin therapy; Grade 4 patients need to be treated with DKA and monitored (17, 37). By the time patients develop clinical symptoms, 80–95% of pancreatic cells are permanently destroyed, so anti-PD-1 treatment can be resumed when glycemic control is stabilized (17, 38, 39).

While anti-PD-1 diabetes can be life-threatening, patients may also have better anti-tumor effects (1, 36). Judd et al. (40) and Morganna et al. (41) both noted that patients with immune-related adverse reactions caused by anti-PD-1 had longer overall survival, longer progression-free survival and higher survival rate.

## Conclusion

As more and more research has confirmed the therapeutic value of anti-PD-1 in malignant tumors, various adverse reactions caused by immunotherapy, such as diabetes, are also increasing. Although the incidence of anti-PD-1 diabetes is low, due to its non-specific symptoms and frequent occurrence of DKA, it may lead to hospitalization or even death, so it must be paid enough attention. In the process of anti-PD-1 treatment, in addition to informing patients of side effects and corresponding symptoms and signs, it is necessary to closely monitor blood glucose and HbA1c before and during treatment, and if necessary, C peptide, diabetes-related autoantibodies, pancreatic exocrine enzymes and pancreatic imaging and even genotypes such as HLA-DR4 can be examined to help diagnosis, in order to identify and treat diabetes earlier and to avoid treatment-related serious adverse events.

## Data Availability Statement

The original contributions presented in the study are included in the article/supplementary material, further inquiries can be directed to the corresponding author.

## Ethics Statement

Ethical review and approval was not required for the study on human participants in accordance with the local legislation and institutional requirements. The patients/participants provided their written informed consent to participate in this study.

## Author Contributions

Z-KZ researched data and wrote the manuscript. Z-GZ revised the manuscript. J-LW, W-XL, T-QW, and M-SC contributed to the treatment of this patient. All authors contributed to the discussion of the article and agree to be accountable for the content of the work. All authors contributed to the article and approved the submitted version.

## Funding

This study was funded by Special fund for clinical research of Wu Jieping Foundation (320.6750.2021-02-76) and Sun Yat-sen University Cancer Center Physician Scientist Funding (No. 16zxqk04).

## Conflict of Interest

The authors declare that the research was conducted in the absence of any commercial or financial relationships that could be construed as a potential conflict of interest.

## Publisher's Note

All claims expressed in this article are solely those of the authors and do not necessarily represent those of their affiliated organizations, or those of the publisher, the editors and the reviewers. Any product that may be evaluated in this article, or claim that may be made by its manufacturer, is not guaranteed or endorsed by the publisher.
